# The perceptions and attitudes of obstetric staff and midwives towards perinatal mental health disorders screening: a qualitative exploratory study in Shenzhen, China

**DOI:** 10.1186/s12912-023-01475-7

**Published:** 2023-09-13

**Authors:** Xiao Xiao, Haixia Ma, Shening Zhu, Qiaomeng Li, Yu Chen

**Affiliations:** 1 Affiliated Shenzhen Maternity & Child Healthcare Hospital, Southern Medical University, Shenzhen, China; 2 School of Nursing and Health Studies, Hong Kong Metropolitan University, Ho Man Tin, Kowloon, Hong Kong China; 3 School of Nursing, Southern Medical University, Guangzhou, China

**Keywords:** Medical staff, Psychological Health Screening, Pregnant women, Qualitative study

## Abstract

**Background:**

The perinatal period is a crucial time for women, as they experience various biological, psychological, and social stressors. Due to the complexity of this vulnerable time, there is a high prevalence of depressive and anxiety disorders among pregnant women. In 2019, the Health Commission of Shenzhen initiated perinatal mental health screening programme in China. However, attitudes and perceptions of medical staff towards implementing mental health screening programme during pregnancy remain unclear. The aim of this study was to explore the perceptions and attitudes of obstetric staff and midwives towards screening for perinatal mental disorders in pregnant women, and identify their perceived needs and motivations for undertaking this task.

**Methods:**

This is a qualitative exploratory study. Data were collected through in-depth, semi-structured, face-to-face interviews. The dataset was analysed using inductive content analysis. Purposive sampling method was used to recruit 13 participants at a tertiary maternal hospital in Shenzhen from September to November, 2019.

**Results:**

A total of 13 obstetric staff was interviewed, including two obstetricians, three midwives, and eight nurses. Four themes were identified from this study: views on perinatal mental health disorders screening, competency in identifying and supporting high-risk groups, barriers to dealing with psychological problems during pregnancy, and the support needs of medical staff in undertaking the tasks of mental health disorders screening.

**Conclusion:**

Medical staff lacked sufficient knowledge and skills in perinatal psychological health and were not well prepared for the task of screening pregnant women for mental health disorders. To address this issue, medical organisations and relevant government sectors should provide training to medical staff on perinatal mental health disorders, enhance public awareness of perinatal mental health disorders, establish a model of multidisciplinary collaboration for the screening of women’s perinatal mental disorders, and provide continuous and holistic care for pregnant women.

## Background

The perinatal period is a crucial time both for women, who will experience biological, psychological, and social stressors [1]. The complexity of this vulnerable time leads to a prevalence of depressive and anxiety disorders for women during pregnancy [2]. It was reported that about 12-13% of women are affected by depression and anxiety disorders during the perinatal period [3]. Mental health disorders in pregnant women may lead to adverse maternal and infant outcomes [4–8], thereby influencing the well-being of the whole family [9, 10].

The National Institute for Health and Clinical Excellence (NICE) in the United Kingdom and the American Psychiatry Association (APA) recommend that all women should be screened at least once for anxiety and depression during pregnancy [3, 11]. Perinatal mental health screening has been implemented in developed countries such as the UK, the United States and Australia, etc. However, only 20-30% of women who were experiencing perinatal mental health disorders were identified or treated due to various provider barriers [11]. For example, many healthcare professionals do not regard diagnosis and treatment of perinatal mental health disorders as their responsibility [12]. Inadequate training, limited access to referrals, treatment and follow up resources; lack of time required for screening; and lack of policy support were all also barriers contributing to complaints from health professionals [13]. Successful implementation and maintenance of a universal screening programme requires additional provider training, increased workloads, and improved perinatal mental health services provided to patients [12]. A warm clinical atmosphere and “normalising perinatal mental health screening” may help to reduce mental health care stigma and enhance women’s acceptance of using mental health care services [13, 14]. The stepped-care model recommended by Miller et al. (2009) suggests that when pregnant women are screened for mental health disorders and referred for treatment within a prenatal care setting, women were likelier to follow mental health care protocols. Therefore, the readiness and preparation of medical staff for such screening would influence the screening effect during the screening process [15–17].

With extensive studies on postpartum depression, postpartum depression screening has been initiated for several years in China. However, anxiety and depression disorders screening during pregnancy have not received enough attention, and no studies related to screening for anxiety and depression during pregnancy have been identified in China. The Mental Health Group in Chinese Preventive Medicine Association (2019) together with the Women’s Mental Health Group in China Maternal and Child Health Association (2019) issued guidelines for screening for perinatal mental health disorders in 2019 [18]. Similar to the guidelines from NICE (2014) [3] and ACOG (2015) [11], the specialist group recommended perinatal mental health disorders screening at least once in the first, second, and third trimester of pregnancy and six weeks postpartum. Edinburgh Postpartum Depression Scale (EPDS), Patients Health Questionnaire-9 items (PHQ-9) were among the most popular tools for depression and anxiety screening [18].

Despite the implementation of the perinatal mental health screening policy, there is limited knowledge on the attitudes of medical staff towards the screening. Therefore, this study aims to explore the attitudes of obstetric medical staff towards perinatal mental health screening. Specifically, the study seeks to understand their perceptions of the screening’s usefulness and necessity, and their beliefs towards the potential benefits and drawbacks of the screening. By gaining a better understanding of medical staff’s attitudes toward perinatal mental health screening, this study aims to identity potential barriers and facilitators to successful implementation of the screening policy and inform strategies for improving perinatal mental health services.

### Research aims and objectives

The aim of this qualitative study is to explore the perceptions and attitudes of medical staff involved in perinatal mental health screening and to explore their perceived needs to provide better perinatal mental health screening services to pregnant women.

## Methods

### Participants and setting

This study was conducted in a tertiary maternal hospital in Shenzhen, located in south China with a population numbering 17.63 million at the end of 2021. The annual birth delivery rate is more than 17,000 in the study hospital and accounts for about 10% of the annual deliveries in the whole city. As a pilot city of the Opening Policy, Shenzhen started to screen for postpartum depression in 2014. Then it initiated mental health disorder screening for pregnant women in 2019. Pregnant women were required to report their mental health status with PHQ-9 in the first, second, and third trimesters of pregnancy. The study hospital plays a leading role in undertaking the task of perinatal mental health screening in this city. Although strategies have been taken to improve the perinatal mental health screening rate, no more than 30% of pregnant women performed the self-report on their mental health status, especially in the second and third trimesters. Therefore, this study was initiated to explore medical staff’s preparation and readiness for perinatal mental disorders screening, and to identify the potential barriers that may have prevented the screening programme implementation.

### Inclusion and exclusion criteria

Purposive sampling was adopted to select participants from a tertiary maternal hospital in Shenzhen from September to November 2019. The inclusion criteria were (1) Obstetricians, nurses, or midwives who directly provide antenatal services to pregnant women and were involved in antenatal mental health disorders screening either at the first trimester, second trimester or third trimester; (2) Had worked in the obstetrics ward or outpatient clinic for at least one year; (3) Were informed about this study and voluntarily participated in interviews. Participants who were on leave due to maternity leave, sick leave, or other reasons during the study period were excluded. In order to obtain a diversity of information, the interviewees included obstetricians, obstetric nurses, and midwives of different ages, working years, and educational levels.

### Ethical considerations

This study obtained ethical approval from the ethics board of the Shenzhen Maternity and Child Healthcare Hospital (Reference NO.2017066). The participants were voluntary to participate in this study and were encouraged to share their experiences freely. The interview place and time were discussed before each interview and determined according to the schedules of the participants. They could withdraw from this study at any time. Written informed consent was obtained from the participants who took part in this study.

### Data collection tools

Participant demographic information, such as age, occupation, years of working, and educational background was collected through a self-constructed questionnaire. According to literature reviews and the research purpose, the interview guide was preliminarily formulated. The preliminary interview questions included: Could you please talk to me about your experience in mental health disorders screening of pregnant women? What is your point of view on pregnant women with mental health disorders? How do you think policymakers and healthcare providers could promote the mental health of pregnant women? Do you have any other questions you would like to elaborate on? After consulting one psychiatric nursing expert, two perinatal psychological experts, one nursing expert in obstetrics, and one midwifery nurse, the question of “How do you identify and deal with pregnant women with mental health disorders?” was added in the interview guide to help understand the level of knowledge and skills of the medical staff.

### Data collection

Semi-structured face-to-face one-to-one interviews were conducted for data collection. Before the formal interview, the interview questions were piloted with several staff. The pre-interviews showed that the interview questions were acceptable and appropriate. In the formal interviews, the interviewer introduced the study purpose to the medical staff who met the inclusion criteria. The interview time and places were decided by the participants. Participant autonomy was assured by obtaining written informed consent.

All interviews were conducted in a quiet, relaxing, and private room. The participants were encouraged to speak freely without interruption. Each interview lasted about 25 to 60 minutes. All interviews were audio recorded, transcribed verbatim in Chinese within 24 hours after each interview. Then translated into English within one week by the first author who obtained her master and doctor degree in English speaking regions. The second author who was both Chinese and English speaker in her work then conducted the backward translation to ensure language equivalency. Field notes were also taken during each interview. Participant confidentiality was promised, with all questionnaires and audio recordings kept in a locked cabinet and only available to the research team. The audiotapes would be destroyed after the research results were published. The data collection and data analysis were conducted concurrently. After each interview, the recordings were transcribed in a timely manner and the transcribed information was analysed. The interview sample size was determined by data saturation when no new information identified from the interviews and the participants repeatedly to express similar ideas on the same interview question. Another two participants were further interviewed to confirm the data saturation had been reached. The interviewer was an experienced obstetric nurse who is familiar with the working practices in the study hospital, but with no relationship to the participants.

### Data analysis

The first author conducted all of the interviews. The data collection and data analysis were conducted concurrently. The inductive qualitative content analysis was used for data analysis [19]. To ensure the transcription accuracy, all transcription content was transcribed verbatim by one author, and double checked by a second author. For data analysis, two authors read the transcription several times to become familiar with the contents of each interview. The first author developed an initial coding scheme by immersing herself in the data and reading the transcribed text word by word. She highlighted the words and phrases from the transcription that captured the different aspects of the interviewees’ perceptions and attitudes towards perinatal mental health disorders screening. The codes were read several times and compared with the context. Then, similar codes were grouped into subcategories to formulate the emerging themes. The two researchers read and coded all transcribed text independently and met regularly to discuss the coding decisions. The research team reached a consensus on the final coding of the themes through discussion.

After the completion of data analysis, the translation of selected quotes was carried out by a bilingual researcher who was proficient in both English and Chinese, and also an expert in women’s health. The translation process was carried out carefully to ensure the accurate conveyance of the original interviews.

### Methodology rigor

To ensure the trustworthiness of this study, all of the interviews were audio-taped and transcribed verbatim. During each interview, key information about the conversation, the nonverbal cues and the situational background were also recorded in the field notes. Field notes were referred to when memories from transcripts of interviews were not clear during the data analysis. Confirmability was assured by data saturation when interviews were continued when no new information emerged from the interviews. To increase the credibility and reduce potential biases, the bracketing strategy was adopted during the data collection and data analysis. In addition, audit trial was conducted to check the accuracy of transcription and data analysis.

## Results

A total of 13 medical staff was interviewed in this study, including two physicians, three midwives, and eight nurses. The average participant age was 34.92 ± 7.28 and the average years of working was 11.15 ± 7.47 years. In terms of educational background, two had graduated from secondary school, three held a college diploma, six held a bachelor’s degree, and the remaining two, a master’s degree (Table 1).

The four themes identified from this study were: Views on perinatal mental health disorders screening, Competency in identifying and supporting high-risk groups, Barriers to dealing with psychological problems during pregnancy, Support needs of medical staff in undertaking tasks of mental health disorders screening (Table 2).

### Views on perinatal mental health disorders screening

The medical staff who were interviewed believed that mental health disorders screening could identify the mental health status of pregnant women at an early stage and provide support for those who may be struggling, thereby reducing the potential adverse outcomes for mothers and babies alike. In addition, mental health disorders screening could enhance healthcare professionals’ professional status. On the other hand, there would be a potential risk of the screening tool generating false positives and false negatives. Healthcare staff must balance between the two choices.

#### Benefits of screening pregnant women for mental health disorders

Routine screening of pregnant women for mental health disorders could help medical staff identify women at high risk of experiencing mental health disorderse.

**Table 1:** Participant demographic information (n = 13)

Coding	Operating post	Age/gender	Professional title	Education background	Marital status	Years of working
P1	Obstetric out-patient nurse	31/Female	Nurse-in-charge	Bachelor	Married	8
P2	Nurse in Obstetric ward	34/ Female	Nurse-in-charge	Bachelor	Married	11
P3	Midwife	34/ Female	Nurse-in-charge	Master	Married	6
P4	Obstetric out-patient nurse	34/ Female	Nurse-in-charge	Bachelor	Married	12
P5	Obstetric out-patient nurse	43/ Female	Nurse-in-charge	Secondary school	Married	23
P6	Midwife	30/ Female	Nurse-in-charge	Diploma	Married	7
P7	Midwife	39/ Female	Nurse-in-charge	Bachelor	Married	19
P8	Obstetrician	55/Male	Chief physician	Bachelor	Married	30
P9	Nurse in Obstetric ward	37/Female	Nurse-in-charge	Master	Married	10
P10	Nurse in Obstetric ward	32/Female	Nurse-in-charge	Diploma	Married	13
P11	Nurse in Obstetric ward	31/Female	Nurse practitioner	Diploma	Married	8
P12	Obstetrician	29/Male	Attending physician	Bachelor	Single	5
P13	Nurse in Obstetric ward	24/Female	Nurse practitioner	Secondary school	Single	5

**Table 2:** Attitudes of healthcare workers on screening for mental health disorders in pregnant women

Themes	Sub-themes
**Views on perinatal mental health disorders screening**	Benefits of screening pregnant women for mental health disorders
	Potential risks of screening for mental health disorders in pregnant women
**Competency in identifying and supporting ‘high-risk’ groups**	Skills in identifying women with mental health disorders
	Approaches to deal with women with mental health disorders
**Barriers to dealing with psychological problems during pregnancy**	Lack of knowledge and confidence
	Working overloaded and fragmented care for pregnant women
	Public stigma towards mental health disorders
**Support needs of medical staff in undertaking tasks of mental health disorders screening**	Strengthen training in perinatal mental health disorders screening
	Enhance public awareness of perinatal mental health disorders
	Establish a multidisciplinary team for the management of perinatal mental health screening
	Development of the perinatal mental health clinic

Early intervention could then be provided to the pregnant women who were identified as having a mental health disorder. This may help reduce the adverse effects of mental health disorders on both mother and infant.

*The benefits of screening are obvious. Screening could help us to identify women with mental health disorders. You know, mental health disorders during pregnancy could have both short-term and long-term effects on mothers and their offspring. When a woman is identified with a mental health disorder, you would pay more attention to her. Early intervention could be provided, and the story could be totally different. (P12)*

In addition to providing benefits to pregnant women, the staff who were interviewed also mentioned benefits to health professionals. Undertaking the task of assessing mental health disorders could expand their role and enhance their sense of competence as a health professional.

*I think screening is a very good experience. By acquiring knowledge and skills, you would pay more attention to the mental health status of pregnant women, which is very important. I could feel personal growth with my role expansion (P4).*

#### Potential risks of screening pregnant women for mental health disorders

Some of the medical staff mentioned that false positives or false negatives when screening was unavoidable. They must be careful when making a decision. If screening with false negatives, medical staff may ignore women who need help and miss the best time to intervene. On the contrary, false positives may increase the stress of pregnant women.

*There are certainly some risks of screening, for example, if a pregnant woman’s mental health disorder could not be identified, resulting in a false negative, she may miss the best time for treatment, while a false positive result is likely to trigger a pregnant woman’s worry, increasing her psychological stress. (P7)*

*I think some women may not pay attention to their mental health status……If they were screened with a false positive result, they may fear being stigmatised. (P13)*

### Competency in identifying and supporting high-risk groups

The competency of medical staff who were interviewed in undertaking perinatal mental health disorders screening was related to their perceived skills and approaches in dealing with women with mental health disorders.

#### Skills to identify women with mental health disorders

The medical staff who were interviewed shared their skills in identifying women with mental health disorders. They would observe their behaviours and facial expressions. By communicating with the women, their intuition would tell them the ones they should pay attention to.

*You could feel that her mood is not right. She would cry. You would notice that her eyes are different from other people. When you talk to her, she is in a daze, or she may have trouble communicating with you. She is obviously in a depressed state. (P4)*

The medical staff said they would also pay more attention to the mental health status of pregnant women based on their experience.

*I would be more conscious when dealing with women with an adverse pregnancy history, such as women who previously had a stillbirth. They may unconsciously be influenced by their unhappy pregnancy history. (P9)*

Medical staff mentioned that an unplanned pregnancy, lack of preparation for pregnancy, and lack of knowledge of normal pregnancy changes and pay too much attention to changes in pregnancy, resulting in anxiety.

*Women with abnormal tests are more likely to be depressed or anxious. If the ultrasound found there might be some problems with the development of the foetus, a woman might fall into a circle of visiting the hospital again and again, consulting physicians repeatedly, and seeking an exact answer from health professionals. (P6)*

The medical staff mentioned that significant family members have a great influence on the mental health status of pregnant women. The amount of social support provided by family members and their perceptions of the pregnancy should also be assessed.

*Some of the old generation is embedded with the traditional belief that “A boy is better than a girl”. Women would suffer from great pressure to “give birth to a boy” to continue the family name. In certain Chinese cultures, some women have to be pregnant seven or eight times to have a boy. I have spoken with a woman who is from Chaoshan district in Guangdong Province. She had four daughters. Influenced by traditional culture and great pressure from family members, she eventually lost interest in everything and fell into a depression. I think the family is very important. (P4)*

#### Approaches to dealing with women with mental health disorders

When pregnant women were identified with mental health disorders, medical staff would use multiple methods to help them, including positive communication and referral to a professional psychiatrist or psychologist.

Communication is a very important skill in dealing with pregnant women with mental health disorders. Medical staff would pay more attention to how they communicated with pregnant women who were assessed as being at high risk of anxiety and depressive disorders.

*The doctors were very careful when communicating with women who were screened within the range of high-risk scores, or those who were taking drugs for a mental health disorder. The doctors would avoid using potentially sensitive words and try to protect the woman’s privacy. (P8)*

Listening and counselling are also very important skills. Medical staff could identify the issues bothering pregnant women by listening to them. Listening to and talking with pregnant women could strengthen the relationship between the women and the medical staff, and allow staff to gain their trust.

*Talking is an effective way for pregnant women to express their emotions. Listening to women could help medical staff understand their concerns and feelings. Hence, we would know what might be helpful. (P1)*

“Non-judgmental care” is important for pregnant women. Treating pregnant women with mental disorders as normal persons is also a strategy for medical staff, who would comfort and give more attention to women with mental health disorders.

*I would treat her as a normal pregnant woman even if I knew she had a mental health disorder. She has the right to be treated equally. If she relied on you, she would cooperate with the examinations. After all, compliance with the prenatal check-ups is very important, both for her and the foetus. (P2)*

Most of the medical staff who were interviewed felt they had inadequate knowledge and skills in terms of dealing with mental health disorders. Therefore, they would tend to refer those women to a professional psychologist or psychiatrist.

*I would advise pregnant women who were screened as being at high risk of mental health disorders to see a psychologist. If symptoms are apparent, it is necessary to see a professional psychiatrist. (P1)*

### Barriers to dealing with mental health disorders during pregnancy

All medical staff who were interviewed believed that psychological health services are very important for pregnant women. However, insufficient knowledge, an overloaded workload, a fragmented nursing model and stigma on the part of the public about mental health disorders are often barriers that hinder screening for mental health disorders.

#### Lack of knowledge and confidence

Most medical staff who were interviewed thought they lacked adequate knowledge and skills. Thus, they felt incompetent in dealing with the mental health disorders of pregnant women. Two of the nurses who were interviewed were screening pregnant women on their mental health status, and said their knowledge in this area is far from enough.

*I felt incompetent with the mental health disorders screening because of my limited knowledge. I am afraid of providing misleading information to pregnant women, although I really want to help them. (P1)*

Some interviewees worried that their lack of skills may potentially be harmful to pregnant women and they could not control the situation when they met with pregnant women with mental health disorders.

*When I evaluate the psychological state of pregnant women, I am worried about my lack of professional knowledge, which will result in some negative outcomes, including poor evaluation, ignoring important symptoms, and using some sensitive words. I may not be able to comfort her unexpected bad emotions and control an out-of-control situation. (P5)*

The medical staff who were interviewed also mentioned they were not clear about the referral procedures. Therefore, they did not know how to guide the pregnant women who were screened as being at high risk of mental health disorders.

*I think the referral system is not complete enough. It makes no sense for high-risk pregnant women if the doctors are unable to provide them with clear guidance. (P2)*

#### *Working overloaded and fragmented care for pregnant women*

The health professionals who were interviewed said they were working overloaded, as if they were working on a production line, and had no time or energy to focus on the mental health status of pregnant women.

*The third child policy has been implemented. At a tertiary maternal hospital, the medical staff is busy with completing routine work. Thus, they have no more time to pay attention to the mental health of pregnant women. (P10)*

Some nurses mentioned that the fragmented model of perinatal care has also restricted access to mental health screening for pregnant women.

*Some pregnant women would have their antenatal examination in different hospitals, and some do not have regular antenatal checkups. Therefore, it is difficult to follow up with women who were identified as being at high risk of mental health disorders. (P4)*

#### Public stigma towards mental health disorders

The medical staff mentioned that many pregnant women regard their mental health status as private and would not talk about their psychological problems.

*Pregnant women are not willing to communicate with us about their innermost thoughts and psychological problems. I think that mental health screening would make them distrust me. Therefore, sometimes I would avoid talking about mental health problems with pregnant women, even if I know something might be wrong. (P2)*

*I thought that as an obstetrician, it was sensitive to talk about mental health issues with pregnant women. I guess that families would assume I was hostile, as if I was judging the women. (P12)*

### Support needs of medical staff to undertake the task of perinatal mental health disorders screening

The medical staff believed that the successful implementation of mental health screening requires relevant policy support from government departments and medical institutions. They hoped to have more opportunity to learn the knowledge and skills required to conduct perinatal mental health disorders screening, more education on perinatal mental health to be provided to the public, and to establish a multidisciplinary team to manage screening and referrals for women with mental health disorders.

#### Strengthen training in perinatal mental health disorders screening

Medical staff suggested that medical institutions systematically train medical staff about pregnancy psychology, to improve their understanding of pregnancy psychology.

*At present, I think our knowledge on perinatal mental health disorders is far from sufficient. Although we may feel the abnormal behaviour of a pregnant woman when we communicate with her, we could not figure out what kind of mental health disorder she was suffering from due to insufficient knowledge in this area. I hope the training would be close to clinical practice. The main purpose of the training is to teach us how to identify and deal with perinatal mental health disorders (P3)*.

*I hope I could be more confident to give suggestions to pregnant women who were screened with a perinatal mental health disorder. I am quite sure that my knowledge of mental health is not enough. Many people told me that I should pay attention to patient mental health, but there was a lack of specific guidance, or professional training to tell me how to work with those women. (P4)*

The health professionals also suggested that hospitals should have clinics with at least one psychiatrist or psychologist to handle perinatal mental health disorders.

*I think women with mental health disorders should be treated by psychiatrists or psychologists. I suggest that each department in the hospital should have a professional psychiatric nurse. Or, we can have a psychology department that can provide perinatal mental health consultation for those women. (P8)*

#### Enhance public awareness of perinatal mental health disorders

The medical staff suggested that the health sector and medical institutions should enhance public knowledge of perinatal mental health and reduce public stigma of perinatal mental health disorders.

*The medical institution should provide more education on perinatal mental health disorders to the significant family members of pregnant women, especially their parents-in-law. Family members should know more about the psychological changes of women during pregnancy, then they could better deal with their negative emotions. Thus, family members would know more about how to take care of pregnant women and their emotions. Maternity schools and online courses are both effective ways. (P7)*

*I think more information on the causes of women’s perinatal mental health disorders and the corresponding coping strategies of pregnant women’s psychological changes should be added to pregnancy schools. (P11)*

#### Establish a multidisciplinary team for the management of perinatal mental health screening

The medical staff suggested the hospital establish a multidisciplinary team for the management of perinatal mental health screening. Such a team could provide continuous care for pregnant women, including screening, referral, and follow-up for their mental health.

*I think perinatal mental health screening should count on a multidisciplinary team, including the pregnant women themselves, and nurses, midwives, doctors, and psychologists or psychiatrists. (P6)*

Multidisciplinary collaboration on perinatal mental health disorders screening involves role clarification, working procedure rules, and guidelines for referrals and interventions.

*If we have a very clear guideline for the team, such as who plays a major role in the screening, and how to refer the women who are assessed with a mental health disorder, I think I would be clearer about my responsibility. (P5)*

#### The development of the perinatal mental health clinic

The medical staff advised that all maternal hospitals should have a perinatal mental health clinic to facilitate the screening of women’s perinatal mental health.

*The screening environment is very important. Otherwise, women may feel nervous and not trust the medical staff. (P5)*

*I think it is better for each hospital to have a perinatal mental health clinic for mental health screening. This could become a practice for antenatal check-ups. (P2)*

## Discussion

### Health care providers lack confidence in psychological health screening during pregnancy

This study shows that medical staff believe that perinatal mental health disorders screening could help identify women with psychological problems. At the same time, it would enrich the professional identity of medical staff and enhance their sense of accomplishment. However, medical staff often felt incompetent in their knowledge and skills in perinatal mental health disorders screening, due to inadequate training in perinatal mental health [16]. They were not confident in using the screening scale and identifying the mental health disorders of pregnant women. Therefore, they were often afraid of misleading the pregnant women and their families by screening them as a false positive or false negative [1] [20–22]. In addition, they do not know how to provide interventions to help women who may potentially be experiencing psychological problems. They were more likely to refer women with mental health problems to psychologists [23]. Thereby, adequate preparation, with relevant knowledge and skills, is recommended before starting to screen for perinatal mental health disorders.

### Factors preventing the implementation of perinatal mental health screening

Perinatal mental health disorders screening is an effective way to identify women with perinatal mental health disorders, with studies in Western countries confirming the significance of perinatal mental health disorders screening [16] [24]. Although the medical staff who were interviewed believed that perinatal mental health disorders screening is very important, there are still many obstacles hindering screening implementation. Due to a shortage of medical staff in most hospitals in China, medical staff are overworked, working as though they are in a production line, and do not have sufficient time to inquire about the mental health status of pregnant women. Such findings were consistent with previous studies in developed countries that have implemented perinatal mental health screening programmes [24]. Therefore, to enable staff to better perform the task of perinatal mental health disorders screening, medical institutions should be equipped with sufficient medical staff who are fully prepared for it [23, 25, 26].

Some of the medical staff who were interviewed mentioned that because of a lack of public knowledge about mental health disorders, the public stigma towards mental health disorders is obvious. Most women still believe that their mental health status is private, and to avoid being labelled, women and their family members would take it as taboo to mention their mental health [27, 28]. This suggests public stigma towards mental health disorders is still a barrier that has impacted the acceptance of perinatal mental health disorders screening. More attention should be paid to enhance public awareness of screening for perinatal mental health disorders. By improving their knowledge of perinatal mental health disorders, pregnant women and their families may have an understanding of the psychological changes that can occur during pregnancy.

## Policy support to better implement perinatal mental health disorders screening

The results of this study show that policy support is a promise for medical staff to more effectively implement the task of perinatal mental health disorder screening. Such support includes a suitable place for mental health disorder screening, and a multidisciplinary team for referral and follow up. At present, there are no standardised guidelines in China for the management of psychological problems during pregnancy. It is suggested that medical staff carry out a systematic evaluation of women’s mental health disorders at different stages of pregnancy [3, 11, 18, 19]. This would optimise the effects of perinatal mental health disorders screening and reduce the potential harms of mental health disorders for mothers and infants alike [16, 28].

### Strengths and Limitations

To the researcher’s best knowledge, this is the first study to explore the perceptions and attitudes of obstetric staff and midwives towards perinatal mental health disorders screening in Shenzhen, China.

There are several limitations in this study. First, the participants were only recruited in one hospital, so the perceptions and attitudes of medical staff in general may be different from the staff interviewed in this study. While it is important to note that this hospital is the largest and most well-established maternal hospital in Shenzhen. In addition, the study hospital plays a leading role in the initiative for the “perinatal psychological health screening programme” in this city. As such, the findings are likely to be representative of the experience and attitudes of obstetric medical staff in similar institution in the region.

Second, the first author’s personal working experiences may potentially have influenced the data collection and data analysis, although the researcher used the ‘bracketing’ strategy during the study process.

## Conclusion

Medical staff highlighted the significance of perinatal mental health screening. However, due to a lack of knowledge and skills in managing mental health disorders, medical staff felt a lack of confidence in undertaking the task of perinatal mental health disorders screening. More efforts should be made to enhance the perinatal mental health training of medical staff and to improve public knowledge of perinatal mental health disorders.

## Data Availability

The dataset generated and analysed during the current study are not publicly available due to the raw data containing information that could compromise the privacy of the research participants and due to the fact that participants of this study did not agree for their data to be shared publicly, but are available from the corresponding author on reasonable request.
